# Factors Associated with Out-of-Pocket Health Expenditure in Polish Regions

**DOI:** 10.3390/healthcare9121750

**Published:** 2021-12-17

**Authors:** Błażej Łyszczarz, Zhaleh Abdi

**Affiliations:** 1 Department of Health Economics, Nicolaus Copernicus University in Toruń, 85-830 Bydgoszcz, Poland; 2 National Institute of Health Research (NIHR), Tehran University of Medical Sciences (TUMS), Tehran 1416833481, Iran; zh-abdi@sina.tums.ac.ir

**Keywords:** health expenditure, out-of-pocket payments, household budgets, Poland

## Abstract

Out-of-pocket (OOP) payments are perceived as the most regressive means of health financing. Using the panel-data approach and region-aggregated data from Statistics Poland, this research investigated associations between socio-economic factors and OOP health spending in 16 Polish regions for the period 1999–2019. The dependent variable was real (inflation-adjusted) monthly OOP health expenditure per person in Polish households. Potential independent variables included economic, labour, demographic, educational, health, environmental, and lifestyle measures based on previous research. A set of panel-data estimators was used in regression models. The factors that were positively associated with OOP health spending were disposable income, the proportions of children (aged 0–9) and elderly (70+ years) in the population, healthcare supply (proxied by physicians’ density), air pollution, and tobacco and alcohol expenditure. On the other hand, the increased unemployment rate, life expectancy at age 65, mortality rate, and higher sports participation were all related to lower OOP health spending. The results may guide national strategies to improve health-care allocations and offer additional financial protection for vulnerable groups, such as households with children and elderly members.

## 1. Introduction

Sustainable financing of health services is a subject of major concern throughout the world [1]. Healthcare spending has typically outpaced economic growth in recent decades in most Organization for Economic Co-operation Development (OECD) countries [2]. While such growth has improved health outcomes, there are concerns regarding the fiscal sustainability of this rising trend, particularly as healthcare systems are predominantly funded from public resources in most OECD countries [3]. Several factors such as demography, rising income, technological progress, and associated healthcare legislation have all been identified as underlying causes behind the persistent growth in health expenditure [4,5].

Health financing involves not only raising adequate financial resources to fund the health system, but also doing so in a way that increases equity and access for all [6]. The main sources of healthcare financing are general taxation, social health insurance, private health insurance, and out-of-pocket (OOP) payments. Over-reliance on OOP spending limits access to care for those who are uninsured or underinsured, undermines health status, deepens poverty, and exacerbates health and socio-economic inequalities not just in low- and middle-income countries but also in high-income ones [7,8]. Empirical evidence highlights that OOP payments are the most inefficient and inequitable means of financing healthcare [9]. The recognized importance of financial protection has led to its inclusion as one of two pillars of universal health coverage, alongside coverage of affordable and good-quality health services [10]. Protecting people against financial hardship associated with using healthcare is crucial for achieving universal health coverage [11].

In Poland, a European Union (EU) member state with a population of 38 million, health insurance contributions (an earmarked payroll tax) are the major source of public healthcare funding, accounting for over 60% of total current spending on health and close to 90% of public health expenditure. Households’ OOP payments were the second-largest source of health financing, accounting for 22.6% of the current spending on health in 2017, more than in most of the developed countries [12]. This implies an increased risk of financial hardship for Polish households. According to a report based on the Poland Household Budget Survey (HBS), 8.6% of Polish households experienced catastrophic health spending in 2014, while 3.8% of households were impoverished or further impoverished due to health spending. Financial hardship is heavily concentrated among the poorest households, with almost 30% of them incurring catastrophic health expenditures [13].

Comprehensive and comparable estimates of health spending in each country are a key input for health policy and planning, and are necessary to support the achievement of national and international health goals as well as to examine a health system’s performance towards financial protection for citizens. A considerable number of studies have been carried out to identify and quantify the determinants of healthcare expenditure at the macro level. The roles of socio-economic, geographic and environmental, lifestyle, and macro-economic factors are well-documented in determining health and healthcare expenditures [4]. However, only a few are concerned with macro-level determinants of OOP spending [14,15], and this financing source might be subject to different economic mechanisms than public or overall private health expenditures. For example, the effects of increased income on health spending might be different for public, private, and OOP spending, and they may have different determinants and follow different patterns as previous evidence has shown [7,16]. This study aims to fill this research gap by explaining the effects of socio-economic factors on OOP health expenditure in Polish regions at the aggregated macro level.

Thus, this paper examined the social and economic factors associated with OOP health expenditures in Poland; for this purpose, we relied on panel-data regression and aggregated household data from 16 Polish regions and for the period 1999–2019.

The paper is organized as follows. This background sets the stage for empirical analysis. The second section provides a literature review on the topic. The third section describes data and methods applied. The fourth section delivers empirical results on factors associated with OOP health spending. The fifth section discusses our results, while the sixth and seventh sections conclude the paper and provide policy implications from our analysis.

## 2. Literature Review on Factors Influencing OOP Health Expenditure

OOP payments for health are known as an inherently regressive source of financing, meaning that poor households face a higher relative burden of OOP payments than households with higher income [8]. Out-of-pocket payments were considered to be the major healthcare financing mechanism in low-income countries, even larger than the government expenditure [9]. The share of OOP expenditure from total health expenditure (THE) tends to decline as government incomes rise and when other forms of financing increase [7]. OOP payments can potentially jeopardize a household’s welfare, leading to catastrophic and impoverishing health expenditures. Such expenditures can reduce people’s ability to spend on other essential goods such as food and clothing, leading to catastrophic health expenditures [17]. Further, households can be impoverished or further pushed into poverty due to OOP health payments. In 2010, almost 808 million people, representing 11.7% of the world’s population, experienced catastrophic spending with OOP payments on health [18], and 122 million people were pushed into poverty [19].

The existing literature on determinants of OOP health expenditure at the micro level (i.e., household and individual levels), has focused extensively on studying the issue in a single country setting, typically using survey data. At the micro level, OOP health payments are defined as “spending on consultations, medicines and tests, and additional payments related to the treatment, and are net of any reimbursement that patients have received or expect to receive from their health insurance programs” [18]. The aim of these studies is to explain inequalities in the distribution of OOP expenditures considering a variety of factors, including household socioeconomic factors, such as income level of the households, employment status, place of residence (rural or urban areas), level of education, age and gender, and gender of the household’s head, as well as factors related to the health status of the household members, usually measured by the number of people with chronic health conditions or disability.

Studies in different countries found that poorer individuals and households have lower absolute OOP expenditures on healthcare than well-off households, but the relative proportion of healthcare expenditure to total expenditures or income is significantly higher among poorer households [20,21]. Households with unemployed heads or those not covered by a social financial safety are at significant risk of incurring OOP payments in different countries [22,23]. Whether the household is residing in an urban area or a remote rural area can influence OOP healthcare expenditure, although the direction of the association can vary. For instance, in Peru, mean OOP spending on healthcare was higher in urban than in rural poor households, which was strongly associated with the near-exclusive use of services delivered by primary healthcare providers [24]. However, in China, health expenditure is higher among the poorest households living in rural areas [25]. In India, rural households paid more than urban households for delivery and neonatal care, regardless of socioeconomic level [26]. In general, existing literature indicates that OOP health expenditures are higher for older individuals, women, and the more educated individuals [27,28,29,30]. Female-headed households are more likely to face a burden of high OOP expenditures than their male-headed counterparts [31], due to having different demographic, sociological, and economic characteristics both in developed and developing countries [32]. Poor health status, disability, and severe or complicated illnesses or conditions often result in higher OOP health expenditures [33,34,35]. For instance, a study conducted in Germany, Belgium, and Czechia found that specific chronic diseases increase the overall OOP burden with a strong association for the medicine burden [33]. Another study performed in Australia concluded that OOP health expenditures are common among senior Australians with chronic conditions, and this expenditure increases with the number of chronic conditions [34].

At the macro level, indicators of macro-economic profiles such as growth in gross domestic product (GDP) and foreign debt, government fiscal capacity, the inflation rate, and unemployment rate have been examined as the most important possible determinants of OOP health expenditures. Applying national health accounts estimates for 191 countries, Musgrove et al. [36] examined the impact of GDP per capita separately on OOP spending and public health expenditure as a percentage of THE. They found that GDP per capita has a significant negative impact on the ratio of OOP expenditures to THE and a significant positive impact on public health spending as a percentage of THE. However, another study by Fan and Savedoff, which examined the effect of factors including per capita GDP, time, government expenditure, and the proportion of population over 60 years old on OOP spending using a dataset for 126 countries from 1995 to 2009, reported that national income had no robust impact on OOP expenditures as a share of THE [37]. Similarly, Grigorakis et al. argued that GDP growth and governmental debt as a share of GDP in OECD and European countries do not have a statistically significant impact on OOP spending [15]. They also reported that governmental debt as a share of GDP does not have a statistically significant impact on OOP spending. However, a study conducted by Xu et al. in 2011 revealed that the increase in external aid for health increased the OOP spending in some lower-middle income countries, which may relate to how external funds are channelled and how they are used in these countries [7]. Fan and Savedoff found that government fiscal capacity appears to be the most significant factor in explaining changes in the OOP share of health spending, indicating that the variation in OOP spending is mainly determined by political and governmental actions [37]. The unemployment rate was reported to have a positive influence on OOP expenditures among selected OECD countries [15]. It seems, despite the universal coverage perspective for health systems in many EU and OECD countries, that unemployed people lose their insurance coverage and thus should deal with OOP expenses in order to meet necessary healthcare needs.

## 3. Materials and Methods

### 3.1. Data

The study used aggregated regional data extracted from an online database of Statistics Poland, Local Data Bank—https://bdl.stat.gov.pl/BDL/start (accessed on 2 December 2021). This database collects regional datasets from a variety of Statistics Poland research, including household surveys, regional accounts, prices, education, population, and environmental issues. The Local Data Bank is Poland’s largest database on the economy, society, and environment, offering more than 40,000 statistical measures thematically grouped. The data are collected for all statistical localities, including gminas, powiats, and voivodships. The advantage of this databank is the cross-regional uniformity of the data collection process and completeness of data for particular variables.

We used the regionally aggregated data, and the regions of interest were voivodships; there are 16 regions of this level in Poland, with a population ranging from 0.9 to 5.4 million people. There were 336 observations for all variables (*n* = 16 regions; *t* = 21 years, 1999–2019) with no missing data; therefore, the panel was balanced. The research period was chosen based on the availability of data; earlier or more recent figures were unobtainable.

### 3.2. Variables

Panel-data regression was used to identify relationships between household OOP health expenditures and a set of socio-economic, health, demographic, and environmental factors. The choice of variables was based on previous literature and the availability of data. The dependent variable was real (inflation-adjusted baselined to 2015) monthly out-of-pocket health expenditure per person in Polish households. The figures used for constructing this outcome measure come from representative household budget surveys with a sample of about 37,000 households, representing 0.3 percent of households in Poland. For the purpose of inflation adjustment in this variable, we used the country-level consumer health price index as reported by the Local Data Bank database. Based on previous systematic reviews, a set of potential independent variables (IVs) was developed, including economic, labour, demographic, educational, health, environmental, and lifestyle measures [4,5]. Real monthly disposable income per person was used to express income, a key measure employed by studies on determinants of health spending [38,39]. Healthcare resources were proxied by the densities of physicians and ambulatory clinics [29,30]. We also accounted for healthcare prices [40,41] by including the price of an outpatient specialist visit. A demographic situation that might affect health spending was proxied by shares of children and elderly in the total population [42,43]. Labour market and educational characteristics included unemployment and employment rates as well as shares of population with tertiary and low education. Health status might also affect health spending and has also been previously used as a proxy for technological progress [40,44]; here, we used the following measures of health: life expectancy at ages 0 and 65, and general as well as cancer- and cardiovascular-related mortality rates. To account for environmental factors, we applied urbanization and air pollutant emission measures [45,46]. Eventually, to account for lifestyle choices, the density of sport club members and expenditures on alcohol and cigarettes were used [44,47]. We did not include any institutional factors in our study because the Polish health system is centralised and there are no region-specific arrangements in health-care delivery. The definitions and descriptive statistics of all candidate variables are shown in Table 1.

In the estimated models, all variables apart from those expressed as percentages were transformed into natural logarithms.

### 3.3. Model Estimation Strategy

We based our model building on study focus, previous research, and statistical properties of models constructed. Stata 14 [48] was used to estimate the models.

Our initial approach was to include variables from all categories of factors potentially affecting OOP health spending as categorized in Table 1 (income, healthcare resources and prices, labour market, demography, education, urbanization, health status, pollution, and lifestyle). However, we did not build a global model including all the potential independent variables, because some of them were highly correlated and described similar phenomena (e.g., *TertEdu* and *LowEdu* with a correlation coefficient of –0.87 and both proxying education level). Instead, we selected one of such competing variables by estimating a separate model with each of these variables included and choosing the one with the lowest Bayesian information criterion (BIC) value. This choice was used for the following sets of variables: (1) *Unempl* and *EmpRate*; (2) *Pop0_4* and *Pop0_9*; (3) *LE_0* and *LE_65*; (4) *Mortality*, *MortCancer*, and *MortCardio*; (5) *Gas*, *Gas-CO2*, *SO2,* and *Nox*. This led to 96 (2 × 2 × 2 × 3 × 4) combinations of IVs and 96 potential initial models.

We estimated one- and two-way fixed effects models (FEMs) in each of these potential initial models to decide whether year dummy variables should be included in the specification (one- and, two-way models include region and, both region as well as year dummy variables, respectively). This was done by testing the hypothesis that the coefficients of all year dummy variables were not different from zero. FEM was used because this approach is appropriate if a focus of analysis is on a specific set of entities and inference is restricted to the behaviour of these units. In contrast, the random-effects model applied if we randomly drew some individuals from a large population [49]. Because here we analysed a complete set of Polish regions, we chose the FEM approach.

Among 96 potential initial models, the model with the lowest BIC value was chosen as a starting point for further backward elimination (which is preferred over forward selection [50]) of IVs. Therefore, the initial model included the following variables (as defined in Table 1): *Income*; *Doctors*; *Clinics*; *VisitPr*; *Unempl* or *EmpRate*; *Pop0_4* or *Pop0_9*; *Pop70+*; *TertEdu* or *LowEdu*; *PopDens*; *LE0* or *LE65*; *Mort* or *MortCancer* or *MortCardio*; *Gas* or *Gas-CO*_2_ or *SO*_2_ or *Nox*; *Sport*; and *TobAlc*. In backward elimination, those variables with *p*-values higher than a chosen threshold of α = 0.1 were excluded one by one, starting from the IV with the highest *p*-value, and the order of exclusion was determined by a decreasing *p*-value. In this step, the FEM approach was also used and the minimization of the BIC value was a criterium to choose the model with a final set of IVs.

This model was also subjected to an analysis of the potential presence of heteroscedasticity, cross-sectional dependence, and autocorrelation, and if any of these were found, remedial estimation approaches were used. Finally, to test the robustness of our estimates, we employed a set of alternative panel-data estimators suitable to our panel structure and potential problems stemming from heteroscedasticity, cross-sectional dependence, and autocorrelation. In particular, fixed-effects regression with Driscoll–Kraay robust standard errors [51,52] was our choice for the base model. In the sensitivity analysis, we applied: (Model S1) Prais–Winstein regression with correlated panel corrected standard errors [53]; (Model S2) a model estimated by feasible generalized least squares allowing estimation in the presence of AR(1) autocorrelation within panels and cross-sectional correlation and heteroskedasticity across panels [54]; and (Model S3) Baltagi and Wu [55] fixed-effects estimator for cross-sectional time-series regression models with first-order autoregressive disturbance terms.

## 4. Results

### 4.1. Dynamics and Variation of Out-of-Pocket Health Expenditure

Table 2 exhibits the real (inflation-adjusted; baselined to 2015) monthly per capita out-of*-*pocket payments for healthcare in Polish regions using 5-year interval figures for the period analysed. The average per capita OOP spending was 43.3 PLN in 1999, and it increased to 58.65 PLN in 2019 with an average annual growth rate of 1.9%. However, this increasing trend was not uniform across the period; the figures show that in 2004 and 2014, real OOP health spending decreased compared to the previous year shown in the table (1999 and 2009, respectively) in several regions. In fact, in 2014 a majority of voivodships were characterised by lower levels of real household health spending than 5 years before.

The analysed health expenditure was fairly diversified across the regions. The coefficient of variation (CoV) for the *Hexp* variable in the period investigated varied from 11.4% in 2002 to 17.8% in 2010–2011, and this variation was notably higher than the respective coefficient for disposable income (from 8.5% in 2018 to 13.1% in 2012; Supplementary File S1). Moreover, the annual average growth rate (AAGR) of OOP health expenditure was noticeably heterogeneous across regions; it reached 2.8% in opolskie region but was only 0.3% in małopolskie (Table 2).

A more detailed analysis of OOP health dynamics is provided in the Supplementary Materials and is discussed in the Discussion section below.

### 4.2. Econometric Results for Determinants of Out-of-Pocket Health Expenditure

Table 3 shows the results of the base scenario (BS) model, which describes the determinants of OOP health expenditure in Polish regions in the period 1999–2019. Because the model built with the strategy applied for the variable selection process (see Section 3.3) suffered from heteroscedasticity, autocorrelation, and cross-sectional correlation, we used a two-way fixed-effects model with Driscoll–Kraay standard errors (results of Pesaran cross-sectional dependence test, modified Wald test for groupwise heteroskedasticity, and Woolridge test for autocorrelation in panel data are shown in the notes of Table 3).

The model was well-fitted to empirical data with adjusted R^2^ = 0.919 (Table 3), and this resulted from a relatively low number of units with enough covariates to explain the variance. Still, it has to be kept in mind that other factors that are potentially significantly associated with OOP health spending were not included in the model. According to the final model, a 1-percent rise in average disposable income (*Income*) was associated with a 0.63-percent increase in OOP health spending. Therefore, according to this finding, healthcare funded by OOP spending is considered a necessary (normal) good. Considering healthcare variables (*Doctors*, *Clinics*, *VisitPr*), only the density of physicians was included in the final specification. A 10-percent increased availability of doctors (*Doctors*) was associated with 1.8-percent higher health spending. No such effect was identified for the accessibility of outpatient clinics. Moreover, our findings show that the price of a specialist private visit did not translate to changes in OOP health expenditure.

The effect of the labour market situation on household health spending is significant but very modest; a 1-percentage point higher unemployment rate (*Unemploy*) was related to 0.01 lower OOP expenditure. On the other hand, demographic variables (*Pop0_*9, *Pop70+*) have a very strong effect on health expenditure; a 1-percentage point higher share of population at age 0–9 (70 and over) would result in 4.9-percent (8.5-percent) increased health spending. Considering population health status, the results are ambiguous. Higher life expectancy at age 65 (*LE_65*) was associated with lower health spending (elasticity of –2.3; in other words, a 1-percent higher life expectancy was associated with a 2.3-percent decrease in OOP health spending), meaning that in the regions where people enjoy longer life, health expenditure is substantially lower. However, when it comes to the mortality variable (*Mortality*), the negative sign of its coefficient was unexpected because greater mortality here is associated with lower spending on health.

Gas pollutant emissions per 1 km^2^ (*Gas*) significantly affected higher OOP expenditure, but this effect was modest (elasticity of 0.07). Finally, the model shows that lifestyle choices were related to household spending on health. The regions characterised by higher expenditures on tobacco and alcohol (*TobAlc*) were the ones where people also spent more on health (elasticity of 0.17). Moreover, a proxy variable for physical activity (*Sport*) was negatively associated with OOP spending; however, this effect was not strong (elasticity of −0.09).

### 4.3. Robustness of Estimates

We applied three other panel data estimators to test the robustness of our econometric estimates: (Model S1) Prais–Winstein regression with correlated panel corrected standard errors; (Model S2) feasible generalized least squares in the presence of AR(1) autocorrelation within panel and cross-sectional correlation and heteroskedasticity across panels; as well as (Model S3) Baltagi and Wu [55] fixed-effects estimator for cross-sectional time-series regression models with first-order autoregressive disturbance terms. The sensitivity analysis shows that the results were fairly robust for the choice of estimator (Table 4).

The elasticity of income (*Income*) was hardly affected by the three alternative estimators (0.60 in S1 and S3; 0.56 in S2, compared to 0.63 in the base scenario (BS)). The coefficient of doctors’ density (*Doctors*) was also similar in sensitivity scenarios (0.15 in all three models) compared to the BS (0.18); however, this variable was only weakly significant in model S3. In general, the results obtained with the Baltagi and Wu [55] FE estimator (model S3) differed from the BS in terms of the significance of some variables. Apart from *Doctors*, *Unemploy*, *Gas*, and *Sport* also proved to be significant only with *p* < 0.1. Moreover, health status (*LE_65* and *Mortality*) and tobacco and alcohol spending (*TobAlc*) were insignificant in this specification. Still, the estimates of the other two sensitivity models (S1 and S2) were very similar to the BS and our findings were clearly supported by these model specifications.

To sum up, the findings of our empirical estimates were stable and presented a coherent picture of factors associated with OOP health expenditure in Polish regions, allowing for comparison with other studies and for drawing policy conclusions.

## 5. Discussion

This research investigated associations between socio-economic factors and OOP health expenditures of households in Polish regions across the period 1999–2019. The study applied the panel-data approach to a variety of variables previously recognized as determinants of health expenditure.

Our analysis shows that the dynamics of real OOP spending varied across years and Polish regions. In the initial years of the period investigated (2000–2002), average OOP spending per capita decreased compared to the previous year in almost all (12–13) of 16 regions (Supplementary Materials). This trend can generally be explained by the dynamics of disposable income, which also declined in a majority of regions, but only in 2001 and 2002. However, for the years that follow, OOP expenditures decreased (year to year) in more than ten regions in 2010, 2014, and 2018, while disposable income declined in these years in only 2–4 regions. Interestingly, the variation in real OOP spending (CoV range: 11.4–17.8%, depending on the year) was relatively high compared to differences in disposable income (CoV range: 8.5–13.1%). The above tendencies suggest that household spending on health was subject to changes resulting not only from fluctuations in disposable income. Moreover, OOP expenditures varied notably in terms of time and spatial dimension. This might reflect the fact that OOP payments put households’ financial stability at risk. A large variation in OOP possibly exhibits its unpredictable character in the form of long waiting times and low private insurance coverage observed in Poland [56,57].

In general, in the period investigated, the average annual growth rate for income was notably higher (3.2% for all regions) than the corresponding growth rate for OOP (1.9%). This suggests that healthcare financed through household spending has characteristics of a necessity (normal good) rather than a luxury. This finding is evidently supported by the estimate of income elasticity from our panel-data regression. According to the model, the income elasticity of OOP health expenditure in Polish regions was 0.63, and this estimate was highly significant and stable in the sensitivity analysis (0.56–0.60). Therefore, Polish households increased their health spending by less than a unit in response to a unit increase in disposable income.

Improved access to health services was previously recognized as a factor contributing to rising healthcare expenditure [5]. When the number of health providers per capita increases, patients have greater access to healthcare services, which may increase the demand for healthcare services. However, an increase in the supply of physicians may lead to the provision of unnecessary services to increase their income, which is called supplier-induced demand [58]. According to our model, the supply of healthcare is associated with higher OOP spending, which may suggest a supplier-induced demand effect. However, it was only physicians’ density that proved to have a significant but modest (elasticity of 0.18) association with households’ health spending; the density of ambulatory clinics was not included in the final model because it was insignificant. Interestingly, the effect of healthcare pricing (proxied by the price of private, fully paid specialist visit) was also irrelevant for OOP; thus, household health spending in Poland seems to be price-elastic. This finding means that when healthcare services become more expensive, households demand less healthcare and keep the OOP unchanged. This result is in line with an American, state-level analysis [45].

In terms of the labour market, a 1-percentage-point rise in the unemployment rate is associated with a 0.01 percent decrease in OOP expenditure; therefore, this effect—although significant—is irrelevant. The linkage between healthcare spending and employment could be explained by the fact that healthcare is not a luxury but a necessity. Similar studies conducted in EU and OECD countries reported that unemployment has a positive effect on OOP spending because unemployed people lose their insurance coverage and thus have to deal with OOP expenses in order to meet necessary healthcare needs [15]. This mechanism, however, does not seem to play an important role in Poland because the unemployed are insured within the public insurance system with no restrictions, while private health insurance is relatively underdeveloped in Poland. Our model suggests that the most important factors behind variation in OOP health spending are those related to the demographic structure of the population. Although previous research was not conclusive about the importance of demographic factors for health expenditure [4], it seems that OOP health spending is much higher in the Polish regions with a higher proportion of children (aged 0–9) and elderly (70+ years) in their populations. Particularly, a 1-percentage point greater share of elderly in the total population is related to as much as an 8.5-percent gain in OOP spending (7.6–7.8% in sensitivity scenarios). The ageing population is expected to contribute to growth in demand for healthcare services and, consequently, greater OOP expenditure since older individuals have more chronic diseases on average and complex health needs. This finding is consistent with similar studies reporting positive age-related influences on OOP payments [30,35]. As the Polish population is ageing, more attention needs to be focused on the healthcare demand and costs of the elderly population. High OOP spending among the elderly in Poland can be attributed to medicines, as almost 60% of household OOP spending is for medicines and medical non-durables [13]. High OOP for medicines closely reflects gaps in coverage caused mainly by user charges but also by waiting times for specialist care. The Poland government introduced an important exemption from user charges for many medicines for people aged 75 and older in 2016. This recent development can be regarded as an important step to protect older people against high medical costs in Poland.

The regression estimates discussed above present a coherent picture; all the coefficient signs are in accordance with a priori expectations. However, the findings are not that clear when it comes to health status measures used in our model. The negative and significant effect of longer life expectancy is expected because if people enjoy better health, they also require less healthcare, and, as a consequence, their resulting OOP spending is lower. This process, however, is not reflected in our estimate of mortality; the coefficient of this variable is negative, despite the fact that we would anticipate it to be positive since higher mortality reflects worse health and, perhaps, more OOP spending. This is not the case here and this contradictory finding is difficult to explain on the basis of only the regression results. This variable might reflect the impact of technological progress on health spending (similarly to other studies, e.g., [59,60]) and the negative coefficient in the model might reflect a decreased need for OOP expenditure resulting from technological improvements. Nevertheless, this explanation is only a speculation and more research is needed to either confirm or reject this hypothesis.

Furthermore, air pollution appears to be associated with higher OOP spending on health. This suggests that poor air quality in Poland [61] leads to increased health needs and, consequently, increased health utilization and spending. In developing countries, environmental factors, such as access to clean water and hygienic sanitation services, housing conditions, air quality, and work conditions are reported as determinants of OOP payments [35,62]. Relevant studies carried out in the OECD have found that air pollution had a significant adverse impact on life expectancy in OECD countries [63], and some studies performed among EU or OECD countries have rigorously estimated its impact on healthcare expenditures [64,65].

Lifestyle behaviours are major determinants of health and accordingly have an impact on healthcare needs and spending. The associations between healthcare expenditure growth and lifestyle-related factors, including alcohol and cigarette consumption, calorie intake, sugar consumption, obesity, and physical activity, were proved by a number of studies performed in OECD countries at the macro level [47,66]. As expected, in our study, the proxy for participation in sports activities was associated with lower household health spending. Finally, higher tobacco and alcohol expenditure were associated with higher OOP, with the elasticity being almost double that of air pollution. Implementing strategies to promote healthy lifestyles could reduce the level of household OOP spending, while also improving population health.

Before we conclude, we shall acknowledge the limitations of our analysis. First, because we used aggregated data, our results are at risk of ecological fallacy, meaning that the relationships observed at the regional level might not reflect household behaviour appropriately. Aggregated data also more often result in confounding bias and cross-level bias. These kinds of bias call for caution in interpreting the findings; however, such an approach is common in the literature, and numerous previous studies based on aggregated data were useful in disentangling relationships between socio-economic factors and health spending. Second, some of the variables we used are imperfect proxies for phenomena they are intended to illustrate: e.g., the price variable (*VisitPr*) used is based on a single price only (private specialist visit), and this definitely oversimplifies the real variation in healthcare prices. Moreover, our outcome variable was based on self-reported spending, making it prone to typical bias associated with survey data, e.g., participants’ inability or aversion to report precise and reliable data. Similarly, life expectancy or mortality rates are also not perfect measures of health status, as they do not explicitly account for morbidity dimension or quality of life. Thirdly, it should be recognized that the results of this analysis were context-specific and their generalization to other settings might not be appropriate. Particularly, Poland has quite a substantial share of OOP spending compared to other developed countries, and this possibly means that the mechanisms behind factors associated with OOP might be different here than in, for example, Western European countries. Furthermore, the present COVID-19 pandemic situation might have important implications for OOP health spending that were not taken into consideration in our study. This limitation results from data constraints; statistics on a set of variables used were not obtainable for the period from 2020 onwards. Therefore, the pandemic effect on OOP spending shall be investigated in the future, once more recent data is available.

## 6. Conclusions

This study provided evidence on factors associated with household OOP health spending in Polish regions based on yearly data from 1999–2019. The roles of socio-economic, geographic and environmental, lifestyle, and other factors are well-documented in determining health expenditures. However, evidence on the impact of such factors on OOP health expenditure at the macro level is still scarce. Our study provided new evidence on the factors affecting households’ OOP spending at the aggregated macro level. Several factors proved to be significantly associated with OOP spending. Our results confirm earlier findings that economic status and demographic structure (i.e., number of children and old aged members) are significant positive predictors of OOP payments. Additionally, we opened a new debate on research into other determinants of OOP payments. Particularly, we identified healthcare (financed from OOP) to be a necessity rather than a luxury good. In addition, we observed a modest supplier-induced demand effect and identified the importance of environmental and lifestyle factors for household health expenditure. Our findings also suggest further research to explore the relationship between health status and OOP spending.

## 7. Policy Implications

One of the main objectives of the national and international health policy is to replace OOP payments with more equitable sources of funding. In this context, analysis of the determinants of OOP payments is of great importance in order to identify problems associated with the accessibility and affordability of healthcare and to devise effective health policies to manage such payments. Based on these findings, several policy implications can be drawn in order to protect Polish households from the negative effects of OOP spending. The study suggests the need for considering a wider range of factors that are positively associated with OOP health spending (i.e. disposable income, the proportion of children and elderly in the population, healthcare supply, air pollution, and tobacco and alcohol expenditure) by policy makers while designing and implementing policies to increase financial protection for the Polish population. The findings can guide national policies toward enhancing allocations to healthcare and providing further financial protection for vulnerable populations, including households with elderly members and children.

## Figures and Tables

**Table 1:** Variable definitions and descriptive statistics.

Variable Definition [Unit]	Mean	Sd	Min	Max
Health expenditure:				
*Hexp*—real ^1^ out-of-pocket monthly health expenditure per person [PLN ^2^]	49.51	10.11	30.08	87.59
Income:				
*Income*—real ^1^ monthly disposable income per person in a household [PLN ^2^]	1192.00	274.71	730.65	2000.40
Healthcare resources and prices:				
*Doctors*—number of physicians per 1000 pop. [person]	2.15	0.33	1.41	2.85
*Clinics*—ambulatory clinics per 10,000 pop. [number]	3.95	1.58	1.01	6.64
*VisitPr*—real^1^ price per outpatient specialist doctor visit [PLN^2^]	78.15	13.47	59.78	119.91
Labour market situation:				
*Unempl*—unemployment rate [percentage]	11.7	5.8	2.1	26.3
*EmpRate*—employment rate [percentage]	63.4	6.3	49.6	78.9
Demographic structure:				
*Pop0_4*—share of population aged 0–4 years [percentage]	5.0	0.4	4.0	6.1
*Pop0_9*—share of population aged 0–9 years [percentage]	10.3	0.9	8.4	13.8
*Pop70+*—share of population aged 70+ years [percentage]	9.6	1.3	6.5	13.1
Education:				
*TertEdu*—share of population aged 15–64 years with tertiary education level [percentage]	17.3	6.4	6.6	38.0
*LowEdu*—share of population aged 15–64 years with lower secondary, primary, and lower education level [percentage]	20.3	5.1	10.8	35.0
Urbanization:				
*PopDens*—population density [persons per km^2^]	129.3	74.8	58.4	388.6
Health status:				
*LE_0*—life expectancy at birth [years]	76.0	1.6	71.7	79.4
*LE_65*—life expectancy at age 65 [years]	16.9	0.9	14.6	18.8
*Mortality*—mortality rate [cases per 100,000 pop.]	987.3	100.6	816.2	1280.8
*MortCancer*—cancer mortality rate [cases per 100,000 pop.]	249.5	26.9	173.9	316.9
*MortCardio*—cardiovascular mortality rate [cases per 100,000 pop.]	446.0	61.4	293.8	639.6
Pollution:				
*Gas*—emission of gas pollutants [tons per year per 1 km^2^]	787.2	836.4	57.1	3751.8
*Gas-CO*_2_—emission of gasair pollutants minus emission of CO_2_ [tons per year per 1 km^2^]	7.02	12.9	0.35	62.8
*SO*_2_—emission of sulphur dioxide [tons per year per 1 km^2^]	2.13	2.76	0.09	16.5
*Nox*—emission of nitrogen oxides [tons per year per 1 km^2^]	1.17	1.27	0.09	6.66
Lifestyle:				
*Sport*—people exercising in sport clubs per 1000 pop. [persons] ^3^	22.5	5.4	9.0	36.4
*TobAlc*—real ^1^ monthly tobacco and alcohol spending per person in a household [PLN ^2^]	29.75	5.67	18.18	47.02

Notes: The number of observations for all variables is 336 (*n* = 16, *t* = 21). ^1^ (a) Health, (b) general, and (c) tobacco and alcohol country-level consumer price indexes were used as a deflator in (a) *Hexp* and *VisitPr,* (b) *Income,* and (c) *TobAlc* variables, respectively. ^2^ PLN stands for Polish złoty; exchange rate (PLN per €): average yearly minimum (maximum) value—3.52 in the year 2008 (4.53 in the year 2004). ^3^ From 2002 to 2020, data were available every two years; missing values were interpolated using linear changes. obs—number of observations; mean—average value; sd—standard deviation; min—minimum value; max—maximum value. Source: Own calculations based on Local Data Bank data (https://bdl.stat.gov.pl/BDL/start - accessed on 2 December 2021).

**Table 2:** Real ^1^ out-of-pocket monthly health expenditure per person [PLN].

Region	1999	2004	2009	2014	2019	AAGR ^2^
dolnośląskie	52.83	46.96	60.25	59.42	67.56	1.5%
kujawsko-pomorskie	34.90	36.31	46.72	44.22	55.48	2.5%
lubelskie	43.75	44.46	48.28	58.08	57.00	1.6%
lubuskie	40.56	40.35	58.48	47.01	58.36	2.4%
łódzkie	50.01	49.38	63.68	60.80	65.10	1.6%
małopolskie	51.99	46.74	55.15	44.63	51.50	0.3%
mazowieckie	57.64	59.85	81.68	71.02	79.77	2.0%
opolskie	41.77	52.49	56.94	52.57	63.68	2.8%
podkarpackie	40.51	44.63	45.47	45.74	47.90	1.1%
podlaskie	34.95	40.97	48.11	49.84	45.37	1.6%
pomorskie	39.04	42.50	53.85	55.79	62.62	2.5%
śląskie	46.15	44.79	48.91	55.57	60.00	1.5%
świętokrzyskie	47.28	38.70	43.98	51.73	65.71	2.2%
warmińsko-mazurskie	34.27	34.12	47.03	45.26	45.28	1.7%
wielkopolskie	38.69	41.89	48.86	43.89	57.17	2.2%
zachodniopomorskie	38.50	41.92	50.61	46.95	55.89	2.2%
Mean value	43.30	44.13	53.62	52.03	58.65	1.9%
Standard deviation	6.82	6.06	9.13	7.41	8.68	0.6%
Coefficient of variation	15.7%	13.7%	17.0%	14.2%	14.8%	-

^1^ Country-level health consumer price index was used as a deflator (baselined to 2015); ^2^ AAGR—average annual growth rate; shaded background in a cell represents a decrease in OOP health expenditure compared to previous year shown in the table (e.g., 2004 compared to 1999). Source: Own calculations based on Local Data Bank data (https://bdl.stat.gov.pl/BDL/start - accessed on 2 December 2021).

**Table 3:** Estimates of panel-data regression model ^1^ describing determinants of real out-of-pocket health expenditure ^2^ in Polish regions in years 1999–2019.

Variable	Coefficient	Standard Error
log(*Income*)	0.631 ***	0.125
log(*Doctors*)	0.180 ***	0.026
*Unemploy*	−0.010 ***	0.002
*Pop0_9*	4.892 ***	1.323
*Pop70+*	8.528 ***	1.918
log(*LE_65*)	−2.310 ***	0.471
log(*Mortality*)	−0.834 ***	0.124
log(*Gas*)	0.073 ***	0.025
log(*Sport*)	−0.085 ***	0.020
log(*TobAlc*)	0.168 **	0.061

^1^ Two-way fixed-effects model with Driscoll–Kraay standard errors was used for estimation (336 observations. *n* = 16; *t* = 21). ^2^ The dependant variable is log(*Hexp*); this variable together with all independent variables are defined in Table 1. log—natural logarithm; ***—*p* < 0.01; **—*p* < 0.05. Adj. R^2^ = 0.919; F-test (model) = 150.3 ***. Diagnostics for the two-way FEM with default standard errors: (1) Pesaran cross-sectional dependence test (CD = −3.11; *p* = 0.002); (2) modified Wald test for groupwise heteroskedasticity in FEM (chi2(16) = 97.11; *p* < 0.001); (3) Woolridge test for autocorrelation in panel data (F = 19.47; *p* < 0.001). Source: Own calculations based on Local Data Bank data (https://bdl.stat.gov.pl/BDL/start - accessed on 2 December 2021).

**Table 4:** Estimates of panel-data regression models illustrating determinants of real out-of-pocket health expenditure in Polish regions in years 1999–2019 using alternative estimators.

Variable	Coefficient (Standard Error)		
	Model S1	Model S2	Model S3
log(*Income*)	0.601 *** (0.101)	0.560 *** (0.057)	0.598 *** (0.120)
log(*Doctors*)	0.149 *** (0.056)	0.147 *** (0.037)	0.145 * (0.079)
*Unemploy*	−0.009 *** (0.003)	−0.009 *** (0.002)	−0.006 * (0.003)
*Pop0_9*	4.653 *** (1.315)	4.186 *** (1.041)	6.469 *** (2.079)
*Pop70+*	7.765 *** (1.757)	7.555 *** (1.160)	7.602 *** (2.343)
log(*LE_65*)	−1.827 *** (0.533)	−1.963 *** (0.300)	−0.592 (0.600)
log(*Mortality*)	−0.621 *** (0.235)	−0.680 *** (0.128)	−0.135 (0.285)
log(*Gas*)	0.066 ** (0.031)	0.076 *** (0.018)	0.067 * (0.036)
log(*Sport*)	−0.084 ** (0.036)	−0.059 *** (0.022)	−0.088 * (0.049)
log(*TobAlc*)	0.134 ** (0.054)	0.143 *** (0.029)	0.067 (0.063)

Notes: Model S1—Prais–Winstein regression with correlated panel corrected standard errors; Model S2—feasible generalized least squares in the presence of AR(1) autocorrelation within panels and cross-sectional correlation and heteroskedasticity across panels; Model S3—Baltagi and Wu [55] fixed-effects estimator for cross-sectional time-series regression models with first-order autoregressive disturbance terms. log—natural logarithm; ***—*p* < 0.01; **—*p* < 0.05; *—*p* < 0.1. Source: Own calculations based on Local Data Bank data (https://bdl.stat.gov.pl/BDL/start - accessed on 2 December 2021).

## Data Availability

This study solely used publicly available data that can be downloaded with no restrictions from Statistics Poland Local Data Bank website (https://bdl.stat.gov.pl/BDL/start - accessed on 2 December 2021).

## Supplementary Materials

The following are available online at https://www.mdpi.com/article/10.3390/healthcare9121750/s1, Table S1: Percentage change of out-of-pocket health expenditure and disposable income in Polish regions in the period 1999–2019.
